# HDL-c levels predict the presence of pleural effusion and the clinical outcome of community-acquired pneumonia

**DOI:** 10.1186/s40064-016-3145-x

**Published:** 2016-09-06

**Authors:** M. Saballs, S. Parra, P. Sahun, J. Pellejà, M. Feliu, C. Vasco, J. Gumà, J. L. Borràs, L. Masana, A. Castro

**Affiliations:** 1 Internal Medicine Department, “Sant Joan” University Hospital (Reus-Spain), Institut Investigació Sanitaria Pere Virgili (IISPV), Universitat Rovira i Virgili, Av/Josep Laporte, 1, 43206 Reus, Spain; 2 Oncology Department, “Sant Joan” University Hospital (Reus-Spain), Institut Investigació Sanitaria Pere Virgili (IISPV), Universitat Rovira i Virgili, Reus, Spain; 3 URLA, CIBERDEM, “Sant Joan” University Hospital (Reus-Spain), IISPV, Universitat Rovira i Virgili, Reus, Spain; 4 Unitat de Medicina Vascular i Metabolisme (UVASMET), Unitat de Recerca de Lipids i Arteriosclerosis (URLA), “Sant Joan” University Hospital (Reus-Spain), Internal Medicine, IISPV, Universitat Rovira i Virgili, Reus, Spain

**Keywords:** HDL, Community-acquired pneumonia, Pleural effusion

## Abstract

**Objectives:**

To investigate if HDL cholesterol (HDL-c) could be a biomarker of the degree of severity according to prognostic prediction scores in community-acquired pneumonia (CAP) or the development of clinical complications such as pleural effusion.

**Methods:**

We included in a retrospective study 107 patients admitted to the hospital that fulfilled diagnostic criteria for CAP between the 30th October 2011 and 1st September 2012. HDL-c levels at admission, CAP prognosis scores (PSI and CURB65) and clinical outcomes were recorded for the study.

**Results:**

Basal HDL-c levels were not statistically different according to prognostics scores neither PSI nor CURB-65. Significantly lower levels of HDL-c were also associated to the development of septic shock and admission to the intensive care unit. HDL-c were inversely correlated with acute phase reactants CRP (*r* = −0.585, P < 0.001), ESR (*r* = −0.477, P < 0.001), and leukocytes cell count (*r* = −0.254, P < 0.009). Patients with pleural effusion showed significant lower levels of HDL-c [28.9 (15.5) mg/dl vs. 44.6 (21.1) mg/dl]; *P* = 0.007. HDL-c is a good predictor of the presence of pleural effusion in multivariate analyses and using ROC analyses [AUC = 0.712 (0.591–0.834), *P* = 0.006]. HDL-c levels of 10 mg/dl showed a sensitivity of 97.6 % and a specificity of 82.4 % for the presence of pleural effusion.

**Conclusion:**

Monitoring HDL-c in CAP is an useful serum marker of acute phase response, clinical outcome and the presence of pleural effusion.

## Background

Community-acquired pneumonia (CAP) is a major public health problem worldwide, with an incidence that ranges from 1.6 to 13.4 cases per 1000 inhabitants per year, a rate that increase with age and comorbidities. CAP is the most frequent infection that causes admission to a hospital. Different studies have suggested that approximately 40 % of patients with CAP are hospitalized and around 10 % of these patients are admitted to an Intensive Care Unit (ICU). CAP is considered to be the leading cause of death among the infections, with a rate that ranges between 2 % in ambulatory patients and 14 % of hospitalized patients. Recent data from the World Health Organization from the 2014 showed that infections of the lower respiratory tract are still the third cause of death worldwide and the second cause of life expectancy lost worldwide (www.who.int/gho/publications/world_health_statistics/EN_WHS2014).

The initial evolution is critical as early clinical failure can occur in up to one-quarter of patients with CAP. Clinical failure is associated with increased complications, length of hospital stay and mortality (Wiemken et al. 2013).

CAP prediction scores were developed to help physicians to define severity of disease and likely clinical outcomes of the patient. Almost all of the major decisions regarding the management of CAP, including site-of-care decisions, revolve around the initial assessment of severity. The Pneumonia Severity Index (PSI) remains the best scoring tool to predict clinical response and long-term outcomes (Fine and Auble 1997; Upadhyay and Niederman 2013; Renaud et al. 2009).

Considering the complexity of the PSI calculation, a great need exists for simple and more accurate scoring tools. Improving these scores will require incorporating additional biomarkers, such as procalcitonin, as well as the research of new biomarkers associated with early and late outcomes (Upadhyay and Niederman 2013).

Another clinical problem not well resolved in pneumonias is to detect those patients that will develop complications such as pleural effusion or empyema. Early detection of those patients is important since a delay in adequate management has prognostic consequences (Falguera et al. 2011).

Recent studies demonstrate that excessive inflammatory response is a major cause of early treatment failure of infections (Fernandez-Botran et al. 2014; Fernández-Serrano et al. 2003; Padrones et al. 2010). Although the inflammatory response represents a defense of the host to the pathogens, this process is beneficial as long as it is limited to the control of local infection. Whenever this reaction is over proportioned, systemic inflammation can give place to serious complications as the disseminated intravascular coagulation, respiratory distress or septic shock that leads to an increase in the morbimortality of these patients (Rittirsch et al. 2008; Sharifov et al. 2013; Steel et al. 2013). Efforts are being made to identify new drugs that can modulate this inflammatory response. Interestingly, one of the drugs under investigation are statins (Takemoto and Liao 2001; McAuley et al. 2014; Criner et al. 2014). Observational studies have suggested that patients who were taking statins at the time of the development of pneumonia were less likely to develop sepsis (Van Lenten et al. 1995), clinical complications or death (Yende et al. 2011; Mortnesen et al. 2012; Viasus et al. 2010). The effect of statins as a coadjuvant therapy in CAP has not been studied, so clinical trials are needed to determine the impact of statins in patients with CAP (Sibila et al. 2013).

Recently it has been discovered that HDL particles possess anti inflammatory, antioxidant and immunomodulatory properties (Norata et al. 2012; Kaji 2013). However, the most studied property and, probably, the best understood is the ability of HDL to promote reverse transport cholesterol from the periphery to the liver for excretion, a mechanism that awards protection against atherosclerosis (Rohatgi et al. 2014). HDL undergoes pronounced structural and functional modifications under acute phase response and inflammation (Navab et al. 2009). When pathological processes such as inflammation overwhelm antioxidant and anti-inflammatory functions of HDL, HDL is converted into a dysfunctional, pro-inflammatory particle (Van Lenten et al. 1995; Navab et al. 2005, 2006; Watanabe et al. 2012; Han et al. 2006).

Clinical studies performed during the course of different infectious have concluded that HDL-c levels were significantly lower in severally ill patients if an infection was present (Kumaraswamy et al. 2012; Cappi et al. 2012; Lekkou et al. 2014; Vermont et al. 2005; Chien et al. 2005). However, there are few data about patients with community-acquired pneumonia (Gruber et al. 2009).

Since there is a relationship between HDL, infection and the acute phase response we wanted to investigate if HDL-c could be a biomarker of success treatment in CAP, the degree of severity according to prediction scores or the development of clinical complications such as pleural effusion.

## Methods

### Study population

This is a retrospective cohort study of patients hospitalized because community-acquired pneumonia (CAP) in a University Hospital. We identified all the patients admitted to the Internal Medicine Department between the 30th October of 2011 and the 1st of September of 2012 with a primary discharge diagnosis of community-acquired pneumonia.

CAP was defined as an acute illness characterized by two or more of the following clinical parameters: fever, chills, cough, sputum production, pleuritic pain, and signs of lung consolidation, along with the presence of an infiltrate on the chest X-ray consistent with the diagnosis.

The inclusion criteria were: over 18 years of age; diagnosis of CAP at discharge; a lipid-profile available by standard laboratory methods in the first routine hospital blood performed at admission.

Patients who did not fulfilled the inclusion criteria or with nosocomial pneumonia were not included.

Medical records of all patients were reviewed. All patients’ medical data and follow-up records were obtained from the inpatient electronic medical records system at the hospital with the permission of the institution according to the institution’s guidelines and ensuring the confidentiality of the patients.

The data included: General patient information; Comorbid conditions; Clinical findings; Vital signs; Blood analyses; Chest radiographs; Microbiology tests; PSI and CURB-65 on admission; Charlson comorbidity index; Clinical evolution during admission; Follow-up after the 30th and 90th day.

### Statistical analysis

Data were processed and statistically analyzed using SPSS statistical package version 20.0 (Chicago, IL, USA).

Normality distribution of continuous variables was checked by Kolmogorov–Smirnov test. Results of continuous variables are expressed as medians and standard deviation in parenthesis. Categorical variables are expressed as percentage. Frequency comparison was performed by Chi square tests. Two-group comparison of normally distributed data was performed by Students t test. For multigrain comparisons, one-way analysis of variance with least square difference for posthoc comparison was applied. Bivariate correlation analyses were performed using Pearson test.

Multivariate analyses were performed to determine factors independently associated with pleural effusion and HDL levels. Results of multivariate analyses are reported as odds ratios with 95 % confidence intervals and P values. We estimated receiver-operating-characteristics (ROC) curve. Sensitivities and specificities for different cutoff values were calculated as well positive and negative predictive values of the model. All testing was two-tailed and *P* values below 0.05 were considered with a statistical significance.

## Results

### General characteristics of study population

107 patients were included in the study. Briefly, the mean age of the patients was 72.64 (16.48) years, which 63.2 % were women. The clinical general characteristics are shown in Table 1.

**Table 1:** General characteristics of the population

N	107
Age (years)	72.69 (16.48)
Gender (men) (%)	63.2
Diabetes mellitus (%)	29
Dyslipidemia (%)	24.3
HTA (%)	40.2
Smoking (%)	19.6
Alcoholism (%)	17.8
Chronic heart failure (%)	17.8
OCPD (%)	45.8
Renal chronic disease (%)	10.3
Clinical presentation (%)	
Cough	73.8
Fever	72.9
Dyspnea	65.4
Pleuritic pain	21.5
Atypical	7.5
Radiologic extension (%)	
None	1.9
Unilobar	77.4
Bilobar	8.5
Bilateral	12.3
Pleural effusion (%)	15.9
Empyema (%)	3.7
SIRS (%)	58.9
Shock (%)	3.7
ICU (%)	6.5
Treatments (%)	
Statins	18.9
Corticoids	68.9
AAS	18.1
Global mortality (%)	2.8
Mortality 30-days (%)	0.9
Mortality 90 days (%)	1.9
PSI	
I	6.5
II	14
III	19.6
IV	39.3
V	18.7
CURB-65	
0	12.1
1	30.8
2	42.1
3	10.3
4	2.8

The more prevalent symptom of the pneumonias included in the study were high temperature (72.9 %), cough (73.8 %) and dyspnea (65.4 %) which are the symptoms of the classical typical pneumonia with crackles (69.2 %) and hypofonesis (25.2 %) in the respiratory exam. The more prevalent radiologic pattern was the alveolar one (95.3 %) with unilobar extension (77.4 %). There was pleural effusion in the 15.9 % of the patients. The more prevalent antibiotics used to treat the patients were cephalosporin (60.4 %) and macrolides (50 %).

Most of the patients included in the study were classified with a number four class using PSI (40 %) and in the number two using CURB-65 (42.9 %) classification. It is noteworthy that although using theses scales there were still an important number of patients that were admitted to the hospital although a low prognostic stratification.

We did not find significant differences regarding HDL-c levels according these prognostics scores neither PSI nor CURB-65.

Using the Charlson comorbidity index we found that most of the patients showed only one comorbidity (34 %) or none comorbidities (12.4 %). Between all the comorbidities the more prevalent was the presence of COPD (45.8 %), hypertension (40.2 %), diabetes (29 %) and dyslipidemia (24.3 %).

We found significant differences of HDL-c levels according to the presence of comorbidities using Charlson index. Interestingly, the lowest levels of HDL-c were in the group of patients without any comorbidity (0 group) with a mean HDL-c of 29.8 (16.6) mmol/dl.

### Mortality rates, follow-up and clinical complications regarding HDL-c levels

We found a low mortality rate in the population of our study (2.8 %). The mortality rates after the 30 days of follow up was 0.9 % and after 90 days was 1.9 %. The number of patients that required a readmission to the hospital after 30 days of follow-up was also very low (3.7 %) and after 90 days there was no any patient.

We did not find any association between HDL levels and mortality rates or the need of readmission to the hospital.

The admission to the intensive care unit (ICU) department was necessary in 6.5 % (n = 7) of the patients. Shock was diagnosed in 3.7 % (n = 4) and also in 3.7 % (n = 4) endotracheal intubation with assisted breathing was necessary.

**Fig. 1:**
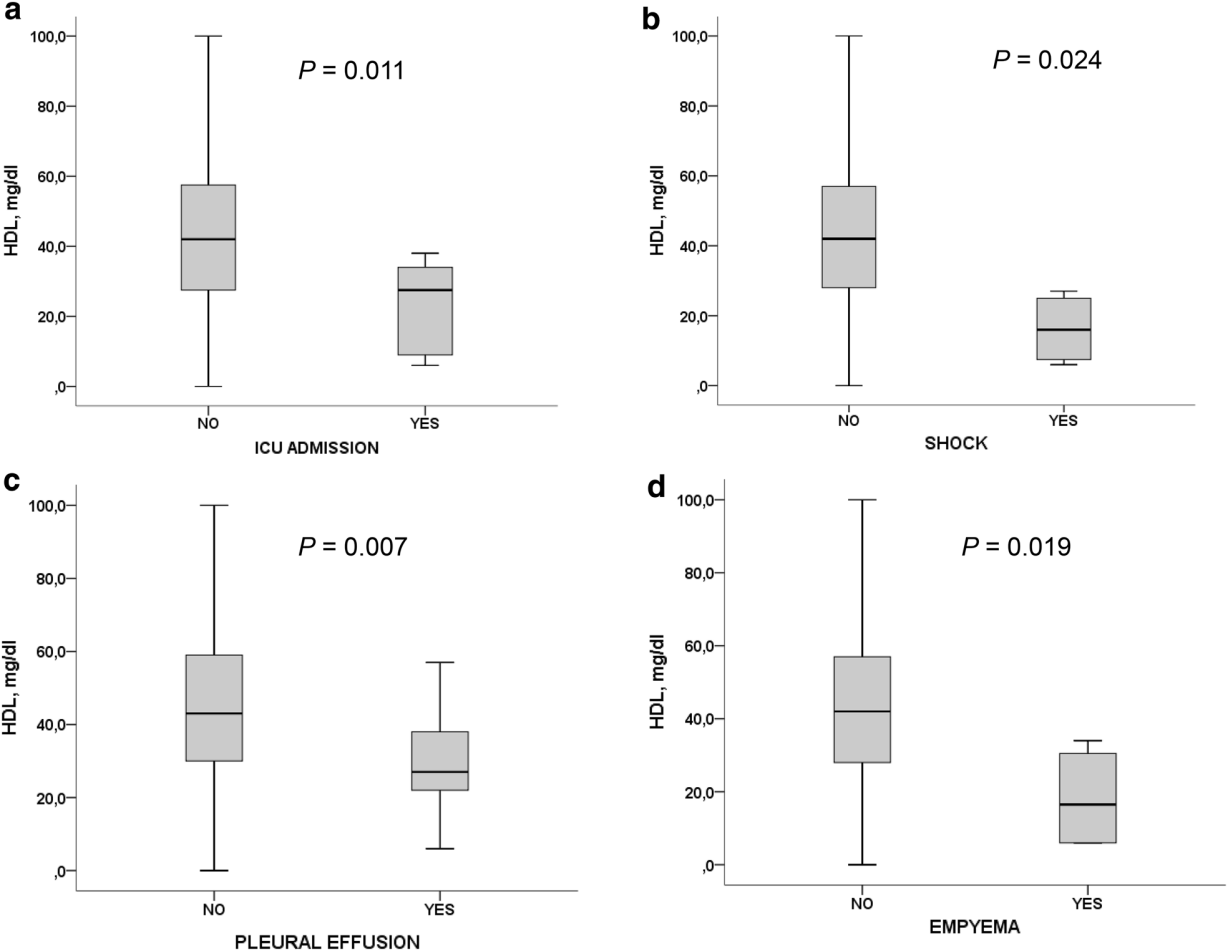
Differences between HDL-c levels regarding the presence of ICU admission, shock, pleural effusion or empyema

In spite of the low number of patients, we found significant differences regarding HDL levels and the presence of shock with a mean of 16.25 (10.3) mg/dl respect 43.3 (20.6), *P* = 0.024 and the admission to the ICU department [23.66 (13.8) respect 43.38 (20.8) mg/dl], *P* = 0.011 (Fig. 1a, b).

### Association between HDL-c and acute phase reactants

**Fig. 2:**
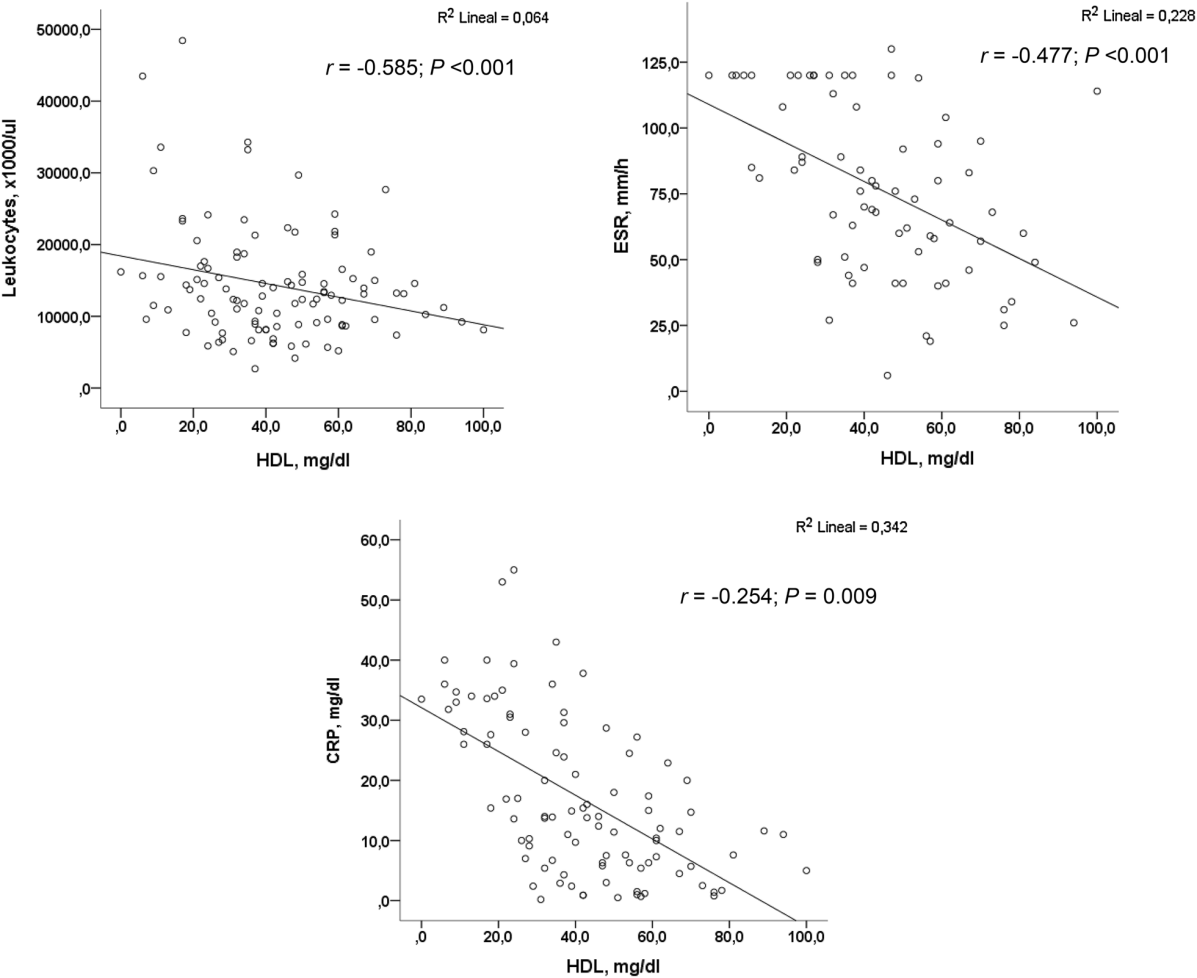
Bivariate correlations between HDL-c levels and leukocyte cell count, ESR and CRP

Bivariate analyses between HDL-c levels and the continue variables showed that HDL-c levels were inversely correlated with the acute phase reactants CRP (*r* = −0.585, P < 0.001), ESR (*r* = −0.477, P < 0.001), and leukocytes cell count (*r* = −0.254, P < 0.009; Fig. 2).

In order to investigate those variables that predicted HDL-c levels in these cohort of patients we included in the linear regression analyses those variables associated with HDL metabolism as age, gender, BMI, TG levels, hypolipemiant treatment and also the clinical variables related to the pneumonia in the univariate analyses with lower levels of HDL-c as the presence of empyema, pleural effusion, shock, ESR, CRP and leukocytes.

**Fig. 3:**
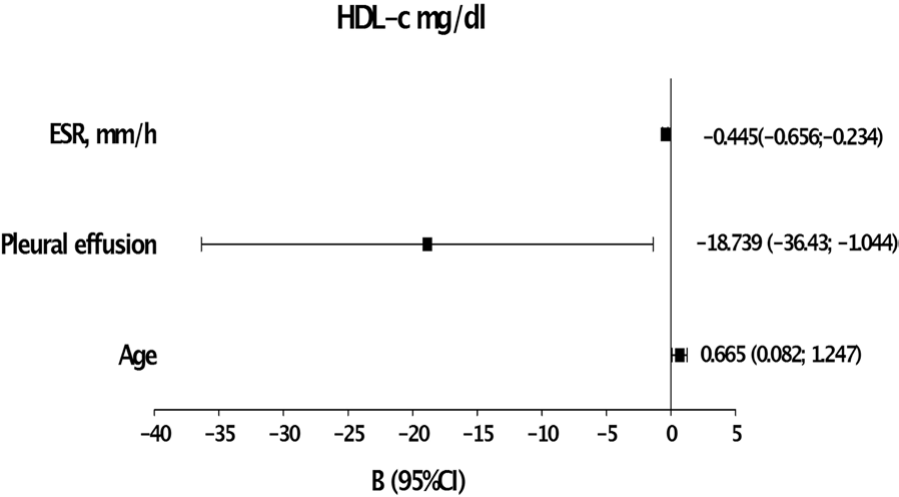
Linear regression analyses. Variables that predict HDL levels in patients with CAP

We found that HDL-c levels were predicted by age [β = 0.665 (0.082–1.247), *P* = 0.028], ESR [β = −0.445 (−0.656 to −0.234), *P* < 0.001] and the presence of pleural effusion [β = −18.739 (−36.43 to −1.044), *P* < 0.039] (Fig. 3).

### Pleural effusion and HDL-c levels

Patients with pleural effusion showed significant lower levels of HDL-c [28.9 (15.5) mg/dl vs. 44.6 (21.1) mg/dl, *P* = 0.007] and in those patients with an empyema there was a stronger decrease of HDL-c [18.25 (14.1) mg/dl vs. 43.2 (20.6) mg/dl, *P* = 0.019] (Fig. 1b, c).

Clinical differences between patients regarding the presence of pleural effusion are described in Table 2. We only found significant differences regarding the presence of pleuritic pain (52.9 respect 15.7 %), higher levels of CRP [25.73 (14.6) respect 15.46 (12.27); *P* < 001] and lower levels of LDL-c [74.47 (20.8) respect 95.88 (28.30); *P* = 0.004]. We did not find significant differences regarding age, gender or the presence of different comorbidities, only a non-significant higher proportion of patients with enolism in those patients with pleural effusion [(35.3 respect 14.8 %) *P* = 0.054].

**Table 2:** Clinical characteristics of the patients regarding the presence of pleural effusion

Variable	Pleural effusion		*P* value
	Yes N = 17	No N = 90	
Age (years)	70.05 (14.7)	73.1 (16.8)	NS
Gender, men (%)	58.8	57	NS
TC (mg/dl)	129.3 (28.0)	165.9 (37.4)	<0.001*
HDL (mg/dl)	29.88 (15.54)	44.6 (21.6)	0.007*
LDL (mg/dl)	74.5 (20.8)	95.8 (28.3)	0.004*
TG (mg/dl)	125.2 (64.4)	126.3 (72.5)	NS
Length of hospital stay (days)	13.2 (6.9)	9.6 (8.4)	NS
SBP (mmHg)	127.5 (41.4)	129.8 (22.7)	NS
Leukocyte cell count (×10^3^/μl)	17.79 (10.78)	13.72 (7.11)	0.051
CRP	25.7 (14.4)	15.5 (12.3)	0.005*
ESR (mm/h)	90.0 (35.6)	74.3 (31.8)	NS
Alb (mg/dl)	4.13 (0.26)	3.55 (0.42)	NS
Pleuritic pain	52.9 %	15.7 %	0.002*
Temperature >38 °C (%)	76.5 %	73 %	NS
Dyspnea (%)	82.4	62.9	NS
Cough (%)	64.7	76.4	NS
Tabaquism (%)	17.6	20.2	NS
Enolism (%)	35.3	14.8	0.054
Diabetes (%)	30.7	23.5	NS
Hypertension	41.2	41.4	NS
Dyslipidemia (%)	29	11.4	NS
OCPD (%)	41.2	47.2	NS
Congestive heart failure (%)	23.5	16.9	NS
Chronic renal disease (%)	17	9	NS
Statins (%)	11.8	19.3	NS
Corticoids (%)	70.6	68.5	NS

* Variables with statistical differences *P* < 0.05

To investigate variables that predicted the presence of pleural effusion we included in the logistic regression model those variables with significant differences regarding the presence of pleural effusion: age, pleuritic pain, HDL-c, triglycerides and alcoholism.

**Fig. 4:**
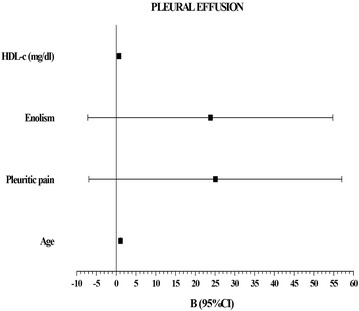
Logistic regression analyses. Variables that predict the presence of pleural effusion

We found that age OR 1.053 (1–1.108), *P* = 0.05; pleuritic pain [OR 11.6113 (2.191–61.553), *P* = 0.001]; alcoholism OR 10.370 (1.815–59.24), *P* = 0.009; and HDL-c levels [OR 0.630 (0.409–0.972), *P* = 0.037] predicted the presence of pleural effusion with a negative predictive value of the model of 98.8 % (Fig. 4).

**Fig. 5:**
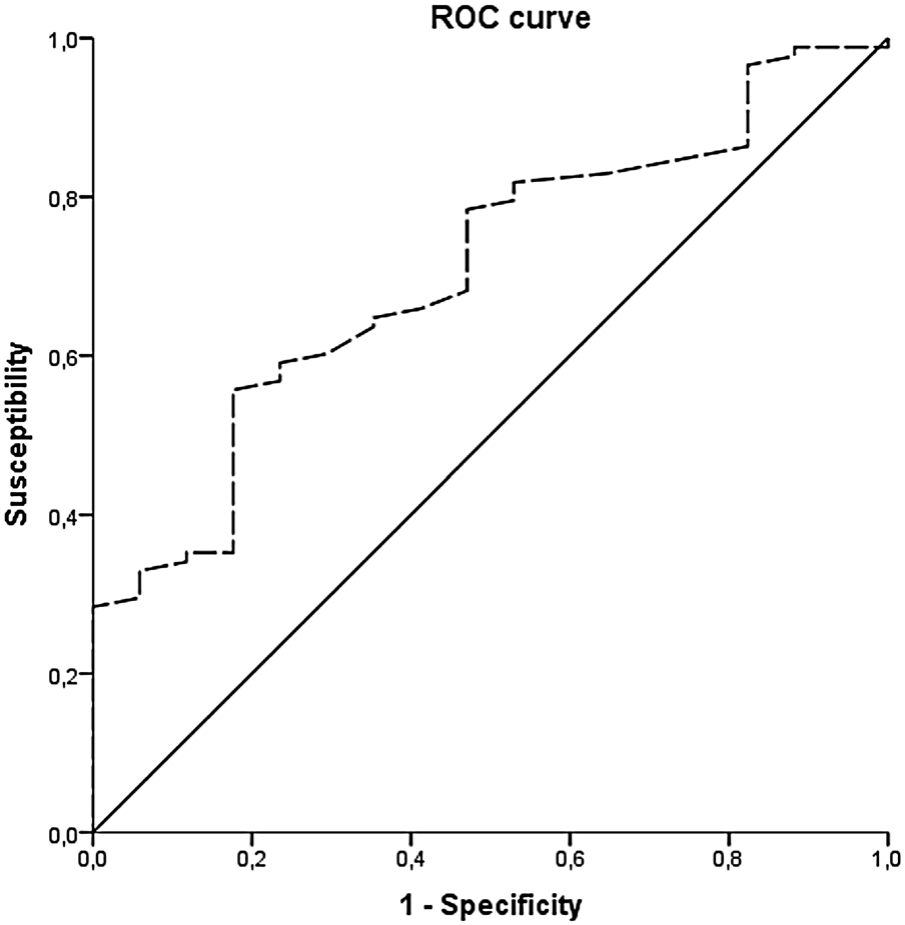
ROC curve. HDL-chol levels predicts pleural effusion

To assess the overall diagnosis accuracy we calculated the receiver operating characteristics (ROC) for each variable included in the model. We found that only HDL-c levels were statistically significant to diagnose the presence of pleural effusion [AUC = 0.712 (0.591–0.834), *P* = 0.006] (Fig. 5). HDL levels of 22.5 mg/dl had a sensitivity of 84.7 % and a specificity of 70.6 %, levels of 10 mg/dl showed a sensitivity of 97.6 % and a specificity of 82.4 %.

## Discussion

In this study we have shown that low HDL-c levels are strongly associated with a worse clinical outcome in patients admitted to the hospital because of CAP. This observation is particularly relevant regarding the risk of hemodynamic inestabilization, the inflammatory response or developing pleural effusion.

We found that those patients with a worse clinical course that made admission to the intensive care unit necessary or presented septic shock showed significant lower levels of HDL-c (Fig. 1). Although there was only a small number of patients included in our study these findings are according to previous results that associate HDL as a prognostic serum marker of infection (Gruber et al. 2009; Nandedkar et al. 2011; Kumaraswamy et al. 2012).

Although it is a retrospective observational study we collected for the study the first lipid profile performed at admission. So we could speculate if low levels of HDL-c are a risk factor for the development of CAP or otherwise they decrease during the infection. The last one seems to be more relievable, since of several studies have shown a decrease of HDL-c levels during sepsis and infection (Vermont et al. 2005; Chien et al. 2005; Gruber et al. 2009). In another observational study in a Swiss cohort of patients with CAP, they also demonstrated a decrease of HDL-c levels during the admission that normalized after the infection (Gruber et al. 2009).

The pathogenic mechanisms to explain how HDL-c levels decrease during infection are not yet well explained. But it is important to consider that HDL particles are composed by several apoproteins that seem to have a pathogenic role during the acute phase response and the innate immune system (Van Lenten et al. 1995; Norata et al. 2012; Kaji 2013; Han et al. 2006; Kumaraswamy et al. 2012; Nandedkar et al. 2011). The results of our study, agree with these previous studies as we confirm this inverse relationship between HDL-c levels and the inflammatory serum markers as CRP, ESR and leukocyte cell count. So, the inflammatory regulatory function of HDL during the infection seems reliable. We have also shown in this study that levels f HDL were determined in the multivariate analyses only by ESR, the presence of pleural effusion and age and not for those variables involved in lipid metabolism.

To understand the pathogenic role of HDL in CAP is important to consider the complexity of HDL function and composition. It could be useful in order to detect those HDL-associated apoproteins that could be potential biomarkers and therapeutic targets for CAP (Sharifov et al. 2013; Han et al. 2006; Guo et al. 2013).

Although, attention the role of HDL or plasma lipids during sepsis and lower respiratory tract infections is increasing (Chien et al. 2005; Gruber et al. 2009; Hao and He 2014; Guo et al. 2013) only few studies were focused on the lipids and the presence of pleural effusion (Vaz 2001; Köktürk et al. 2005).

The results of our study show that levels of HDL-c could be a good biomarker of pleural effusion in patients affected by NAC. A level of HDL-c lower than 10 mg/d showed a sensitivity of 97.5 % and a specificity of 82.4 %. The evolution of hospitalized patients with CAP in the first or 3 days is crucial, it is important to detect the presence of pleural effusion or the patients at risk of it because of the optimal treatment approach is important for the course of infection that may include an early thoracocentesis or surgical drainage of empyema. For this reason we think that determination of HDL-c could be easy and useful in the decision-making in the ordinary clinical practice.

It was proposed in few studies that increased permeability of pleura during infection allows the pass of lipids from serum to the pleura and they could be useful to discriminate transudate respect exsudate effusion (Kumaraswamy et al. 2012). These could explain the decrease of HDL and LDL in the plasma that we found in those patients with pleural effusion. But, as we found a strong inverse relationship between HDL-c and the inflammatory serum markers as CRP, leukocyte and ESR we can elucidate that HDL maybe is not only a specific serum marker of pleural effusion (transudate or exsudate) but it is a good serum marker of the increased systemic inflammatory response of these patients and the organ-specific inflammation, in this case, the pleura.

This is a retrospective study so there are some important limitations. We have a selection bias because we only included those patients affected by CAP admitted into the hospital and we don ‘t have data about those patients treated in-home or those that were discharged at home from the emergency department. But the results seem to be relevant. On the other hand, most of the patients included in this study showed the typical clinical presentation so misdiagnosis rate seems to be low.

We conclude that the monitoring of HDL-c levels in CAP could be useful as serum marker of acute phase response after infection and the presence of pleural effusion. Clinical course of CAP is not only influenced by the etiologic agent but also influenced by the host inflammatory response. This may be associated with the clinical course of the patient and the organ involvement. HDL could add information guiding antibiotic therapy or to decide when to admit a patient affected by CAP into the hospital or the risk of pleural effusion and empyema.

## Abbreviations

CAP: community-acquired pneumonia
HDL-c: high density lipoprotein cholesterol
